# The Applicability of Acceptance and Commitment Therapy for Obsessive-Compulsive Disorder: A Systematic Review and Meta-Analysis

**DOI:** 10.3390/brainsci12050656

**Published:** 2022-05-17

**Authors:** Tamini Soondrum, Xiang Wang, Feng Gao, Qian Liu, Jie Fan, Xiongzhao Zhu

**Affiliations:** 1 Medical Psychological Center, The Second Xiangya Hospital of Central South University, Renmin Middle Road 139#, Furong District, Changsha 410011, China; tanesha0510@hotmail.com (T.S.); xiangwangpsy@csu.edu.cn (X.W.); psychgf@163.com (F.G.); 208201004@csu.edu.cn (Q.L.); fine1025@126.com (J.F.); 2 Medical Psychological Institute of Central South University, Changsha 410011, China; 3 National Clinical Research Center for Mental Health Disorders, Changsha 410011, China

**Keywords:** obsessive–compulsive disorder, acceptance and commitment therapy, psychological inflexibility, meta-analysis, systematic review

## Abstract

Background: Acceptance and commitment therapy (ACT), a third-generation cognitive behavioral therapy (CBT), has proved its efficacy amidst various mental disorders. A growing body of studies has shown that ACT can improve obsessive-compulsive disorder (OCD) severity in recent years. To assess the effect of ACT on OCD, we carried out a systematic review and meta-analysis to provide a basis for therapists to use different psychological dimensions of ACT for OCD. Methods: PubMed, the Cochrane Library, EMBASE, EBSCO Host, and literature references were searched until May 2021. Randomized controlled trials (RCTs) and other study designs assessing the effect of ACT among adults suffering from OCD were examined. Results: Fourteen studies, including 413 participants, published between 2010 and 2021 were identified. ACT made statistically significant progress in the Yale–Brown Obsessive–Compulsive Scale (YBOCS) compared with control conditions. Conclusion: After reviewing all the ACT studies, we acknowledge the plausibility of ACT in treating OCD and improving its symptoms for the clinical population. ACT can also be an adjunct therapy for other well-established treatments. It also favors targeting psychological inflexibility. Further well-controlled and high-quality RCTs are required for a better conclusion in further studies.

## 1. Introduction

Obsessive–compulsive disorder (OCD) is a neuropsychiatric disease characterized by recurrent obsessions and compulsions [1]. OCD has an estimated prevalence of approximately 2%, and according to the World Health Organization (WHO), it has been positioned among the 10 most incapacitating illnesses [2]. As such, if OCD patients do not seek treatment, they tend to experience functional impairment and reduced quality of life [3]. Considerable improvement can be attained in many patients, but for about 50%, the treatment response is incomplete (Fineberg et al., 2015) [4], which emphasizes the need to develop and promulgate effective treatments [5].

Recent studies have thoroughly assessed the effectiveness of pharmacotherapy for OCD and validated the use of selective serotonin reuptake inhibitors (SSRIs) and clomipramine (tricyclic antidepressant [4,6,7]. With regard to psychotherapies, American Psychiatric Association (APA) endorses cognitive behavioral therapy (CBT) with exposure and response prevention (ERP), along with SSRIs, as safe and effective primary treatments for OCD. Psychological treatments that use exposure are currently considered the most effective form of treatment for OCD and are associated with a 60–85% reduction in symptoms [8]. Indeed, exposure and response prevention (ERP) for OCD acts as an organized and manualized approach to psychotherapy and has shown superiority to other psychological treatments. Exposing OCD patients to feared stimuli and simultaneously helping them abstain from carrying out compulsions has proved revolutionary. Presently, ERP is considered the gold standard psychotherapy for OCD [9]; however, it bears some limitations.

Owing to the drawbacks of ERP, there has been an impending need to seek alternatives and reinforce them. Third-wave behavioral therapies, such as acceptance and commitment therapy (ACT), have become more popular over the past years [10]. ACT emphasizes the willingness to experience unwanted thoughts and emotions and connect with the present situation rather than divert the emotional impact. Therapeutic procedures applied in ACT are rooted in three central philosophical bodies: functional contextualism, applied behavioral analysis, and relational frame theory (RFT) [11,12,13]. These three bases of ACT purport that people learn how to shift the functions of stimuli to other stimuli using various frames such as time, space, causality, and perspectives. For example, a contamination-type OCD experiences “dirty” things as dangerous, and anything related to “dirty” will be labeled “dangerous”. The behavior will be inconsistent with environmental contingencies and rather influenced by cognitive contingencies. As such, a suppressed response will resurface in the appropriate emotional and environmental context [14,15]. Therefore, ACT aims at addressing cognitive contingencies by not focusing on contents or emotional controls but instead accepting those thoughts and feelings without the necessity to act upon them. If the patients continue to experience those thoughts and feelings without responding to them, they will not meet the criteria for OCD.

Researchers have come up with six areas of focus in ACT [16,17] that englobe different types of psychological processes, namely acceptance, defusion, the self as context, contact with the present moment, values, and committed action, all of which support behavioral flexibility. ACT works by developing psychological flexibility and accepting intrapersonal conflicts, and more importantly, ACT focuses on mindfulness and maneuvering around unpleasant thoughts [18]. Innumerable studies have demonstrated that alterations in psychological flexibility, valued living, and mindfulness complement the clinical prognosis among OCD patients. Therefore, the main goal of ACT is not to resist or reduce the number of obsessions and compulsions but rather to teach patients to alter their rapport with obsessive thinking so that they do not wrestle against these occurrences [19]. Obsessions and compulsions will be seen as merely “thoughts that rise and fall” and part of the complexities of life. ACT also aspires to overcome the weaknesses of ERP; if the patient is resistant to complex exposure maneuvers, the combination of ACT procedures may help in promoting treatment participation [20]. Nevertheless, ACT may be carried out in other ways; for example, one study has successfully implemented ACT without any in-session exposure exercises [17]. Considering these theoretical foundations of ACT for OCD, it will be interesting to see how those theories have been implemented across studies.

Previous systematic reviews and meta-analyses were conducted on ACT for anxiety and OCD spectrums and various other disorders. It was concluded that ACT was “possibly efficacious” in treating OCD [21]. The most recent review on ACT for OCD found enough evidence to encourage the use of ACT when combined with pharmacotherapy [22]. Based on this recent review, we are currently aiming at (1) assessing the effect of ACT on OCD, (2) understanding the psychological mechanism and the development of psychological flexibility in ACT for OCD, and (3) alluding to the dodo bird hypothesis. To the best of our knowledge, the present study is the first comprehensive systematic review with a meta-analysis relating to the use of ACT for OCD. The data comparing ACT to well-defined treatments such as SSRIs and ERP were also assessed. Several studies have reported significant changes in OCD symptoms after ACT interventions, while some have found no significant changes, and these inconsistencies validate the need for the present study. The main goal of this review and meta-analysis is to assess whether ACT can be a viable full-fledged therapy for OCD or adjunct therapy to well-defined treatment and facilitate changes in psychological processes distinct from CBT.

## 2. Materials and Methods

### 2.1. Data Sources and Selection of Studies

Literature searches were executed in PubMed, the Cochrane Library, and EMBASE, comprising studies published until May 2021. The grouping of keywords used was acceptance and commitment therapy AND (obsessive-compulsive disorder OR OCD OR compulsions OR obsessions). Those search terms were employed in changeable versions to look for supplementary literature on Google Scholar. The 95 results were then assessed for pertinent materials to decrease publication bias. Two reviewers screened the titles and abstracts. Eligibility criteria adhered to the PICOS framework [23].

Participants. The participants were adults aged 18 years or older. All participants were diagnosed according to DSM-V criteria for OCD.

Interventions. One primary approach, acceptance and commitment therapy, was reviewed.

Controls. Both active (pharmacotherapy, progressive relaxation technique, exposure–response prevention) and inactive control conditions were considered.

Outcomes. Obsessive and compulsive-related data should be shown at both the baseline and the posttreatment. The changed scores from baseline to posttreatment in YBOCS and YBOCS-SR were noted to ascertain OCD severity [24]. It should be noted that the YBOCS-SR produces similar scores to the clinician-administered version of the YBOCS and reveals good internal consistency [25,26].

### 2.2. Extraction of Data and Assessment of Study Quality

Two reviewers impartially vetted the titles and abstracts of all the studies generated from the retrieval to analyze if they qualified for review. The next step was to obtain full texts and assess them based on predetermined eligibility criteria. If the reviewers encountered any divergence, the third reviewer was meant to settle the issue by examining it alongside. The data were retrieved using forms of data extraction, which were directly designed. The first reviewer (T.S.) extracted the information based on the structured forms; the second reviewer (X.W.) verified their accuracy and extensiveness. The extracted information comprised the author(s), publication year, participant characteristics, frequency, types of intervention, dropout rates and duration, outcome measurements, and the primary outcomes. The Engauge Digitizer 10.4 (Version 10.4 (10 October 2017, Mark Mitchell, Torrance, CA, USA) extracted the data only if figures were presented [27]. The Cochrane Risk of Bias tool was employed to evaluate the risk of bias, including selection bias (random sequence generation, allocation concealment), attrition bias (incomplete outcome data), detection bias (blinding of outcome assessment), performance bias (blinding of participants and personnel), reporting bias (selective outcome reporting), and other types of bias [28]. Each element was considered as high, unclear, or low risk.

### 2.3. Statistical Analysis

Stata version SE/12.0 was used for the analysis of data. Due to participants’ different baseline values, we employed changed scores (from baseline to posttreatment) to estimate standardized mean differences (SMDs) and 95% CIs. The universal estimation being r = 0.5 was used as the correlation coefficient amid posttreatment and pretreatment totals [29]. The scale of the SMDs specified the following: 0–0.2 means negligible effect, 0.2–0.5 means small effect, 0.5–0.8 means moderate effect, and 0.8+ means significant effect, according to Cohen (1988). We estimated heterogeneity with the I-squared statistic. The randomized effect model should be applied if I^2^ ≥ 50% or the *p*-value is less than 0.1, which would signify remarkable disparateness. Subgroup analyses were operated in line with the two groups (inactive or active control conditions).

## 3. Results

### 3.1. Search Results

**Figure 1:**
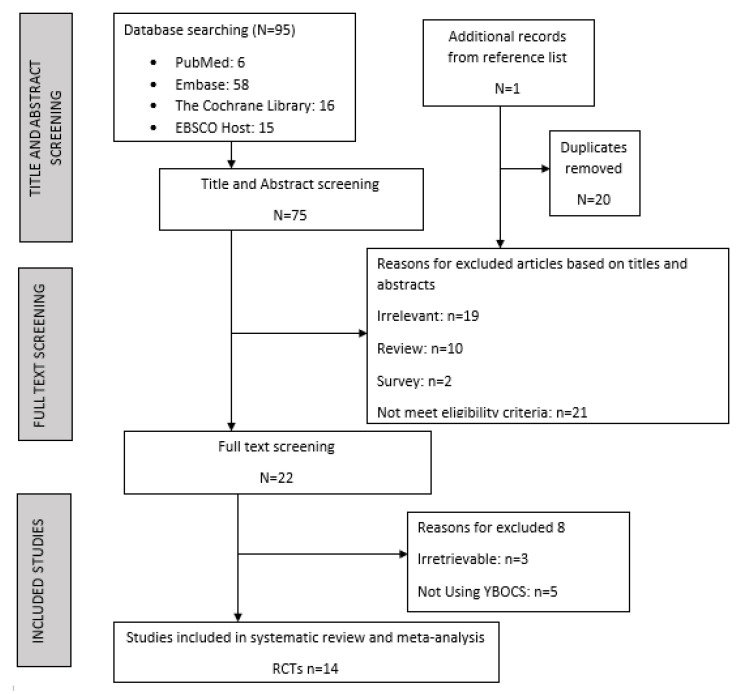
Flowchart of trial selection process according to PRISMA.

Altogether, 95 possibly pertinent records were extracted (6 from PubMed, 58 from EMBASE, 16 from the Cochrane Library, and 15 from EBSCO Host). After excluding duplicates, we reduced the significant records to 75 and omitted 53 from the review for diverse reasons. Within the 22 full-text manuscripts measured for eligibility, 14 were kept, including 1 article from the reference list. In the end, 14 studies consisting of 413 participants were included in the meta-analysis and systematic review. The meta-analysis was conducted with 8 studies due to 5 being case reports and 1 RCT having insufficient data. Figure 1 gives a detailed summary of the selection process.

### 3.2. Characteristics of Included Studies

Table 1 summarizes the characteristics of the studies included and the 14 studies published between 2010 and 2021. Participants are clinically diagnosed OCD patients with a mean age of 19 to 40 years. The interventions covered ACT with other therapies such as ERP, narrative therapy, and pharmacotherapy. The comparisons included placebo, no intervention, other therapies, and pharmacotherapy. The intervention duration differed from 3 weeks to 20 weeks.

### 3.3. Research Evidence on ACT for OCD

To date, there are around 300 RCTs for ACT, analyzing various conditions, and few have found ACT to be an effective treatment for OCD. Several meta-analyses have found optimistic effect sizes for ACT in treating various mental illnesses. Currently, nine RCTs exploring the role of ACT for OCD have been conducted.

### 3.4. Risks of Bias of Included Studies

**Figure 2:**
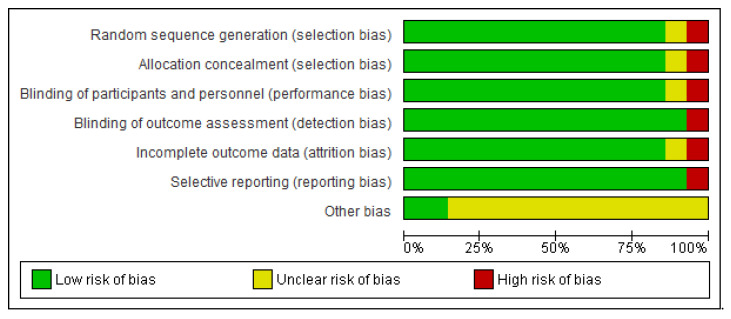
Risk of bias graph: review authors’ judgments about each risk of bias item presented as percentages across all included studies.

**Figure 3:**
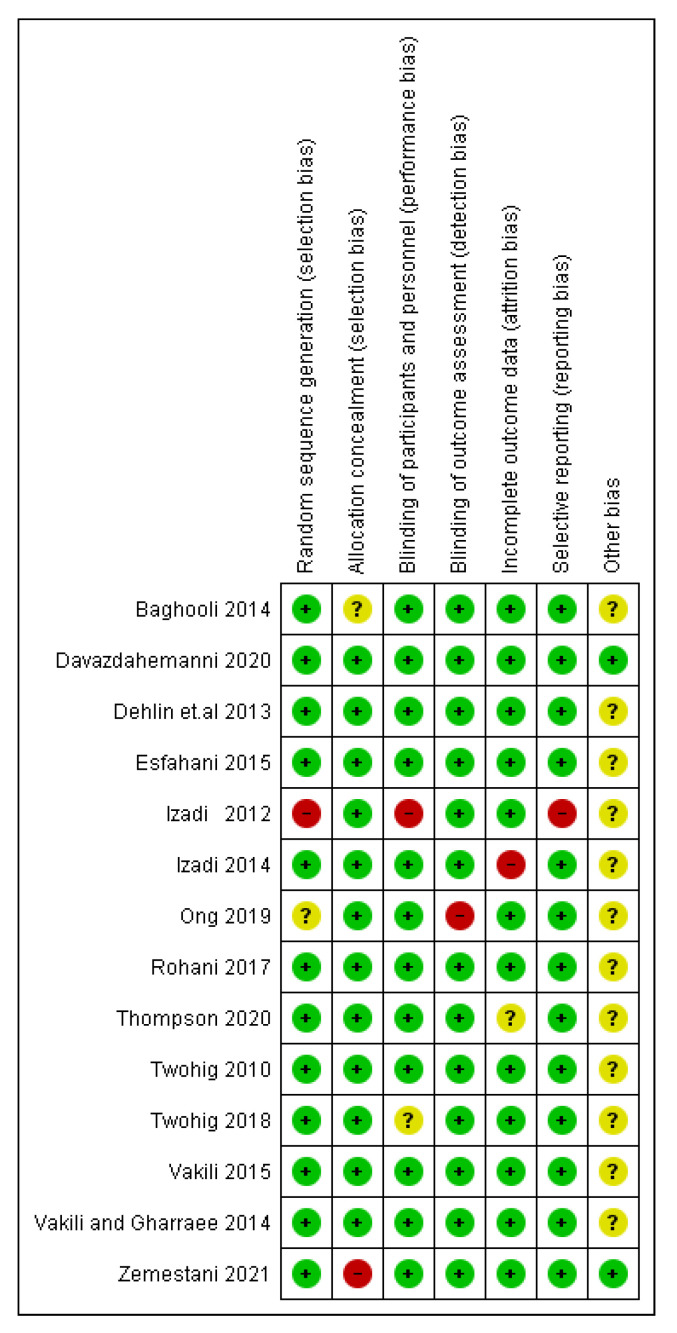
Risk of bias. + = high quality, − = low quality, ? = unclear. Note: The above studies are included in the reference sections; Twohig (2010) [30], Izadi (2012) [31], Dehlin et al., (2013) [32], Vakili and Gharraee (2014) [33], Baghooli (2014) [34], Izadi (2014) [35], Esfahani (2015) [36], Vakili (2015) [37], Rohani (2017) [38], Twohig (2018) [39], Ong (2019) [40], Thompson (2020) [41], Davazdahemanni (2020) [42], Zemestani (2021) [43].

Figure 2 and Figure 3 describe the analysis of the risks of bias. No study was entirely evaluated as bearing a low risk of bias through all the study areas. The random sequence generation and adequately concealed allocation had a low risk (12 studies or 90%). A few studies did not state if blinding techniques were used, perhaps because the authors presumed that blinding was not viable due to the intervention’s quality. Regarding the blinding of participants and personnel, 12 studies were reported to have low risks and 1 study was reported to have unclear and high risks.

Regarding blinding the outcome estimations, 13 studies (95%) were evaluated as bearing low risks as objective measures rarely gauge the outcomes. As for the incomplete outcome data bias, 12 studies (90%) bear low risks because they conveyed a low rate of dropouts, 1 study was found to have an unclear risk, and 1 study was found to have higher risk. The selective reporting bias was presented as low risk, with 95% of all the studies if all stipulated outcomes were reported. Mostly, every study had an unclear risk of bias under the other bias criterion, and two studies were determined as low risk.

### 3.5. Meta-Analysis Results

In the present meta-analysis, a few included studies failed to mention the follow-up effects, and the follow-up periods were also varied. Hence, our meta-analysis is meant to assess the instant postintervention effects of ACT. Table 2 demonstrates the overall effects of ACT on the YBOCS. All 8 studies, totaling 366 participants, used the YBOCS to evaluate the ACT effects on OCD symptom reduction. A significant heterogeneity (I^2^ ≥ 50%) was found; thus, we employed the random-effects model. The results confirmed that the intervention group has a statistically significant overall effect compared with various conditions (effect size: −1.19; 95% CI: −1.87 to −0.51; *p* < 0.000), with an I^2^ of 87%. ACT demonstrated an overall effect that is statistically significant compared to control conditions.

### 3.6. Subgroup Analyses

**Figure 4:**
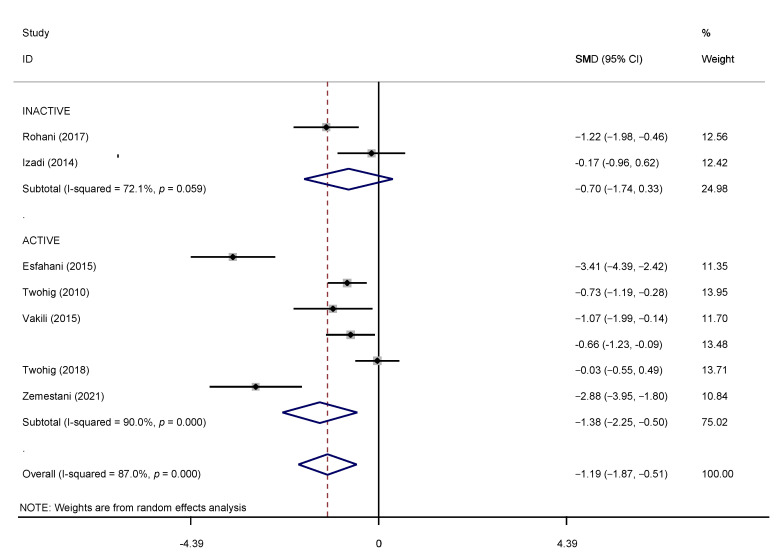
Forest plot of effect estimates of ACT versus controls on YBOCS. Note: The above studies are included in the reference sections; Twohig (2010) [30], Baghooli (2014) [34], Izadi (2014) [35], Esfahani (2015) [36], Vakili (2015) [37], Rohani (2017) [38], Twohig (2018) [39], Zemestani (2021) [43].

Based on the results above, a subgroup analysis was necessary due to the impediment caused by the active control interventions. Despite a small sample, we noticed the efficacy of ACT in reducing obsessive–compulsive symptoms when paralleled with inactive control groups. The results of the subgroup analyses portrayed various statistically significant effects for active control conditions and non-significant effects for inactive conditions. They are as follows: −1.38 (95% CI, −2.248 to −0.508; *p* < 0.000) with a 90% I^2^ for active control and −0.702 (95% CI, −1.735 to −0.332; *p* < 0.059) with an I^2^ of 72.1% for the inactive control condition, all explicitly shown in Table 2 and represented by a forest plot in Figure 4.

## 4. Discussion

### 4.1. Main Findings

The overall effects of ACT in reducing OCD on the YBOCS parameter were statistically significant, with a large effect size. It is worth noting that the types of control conditions might have influenced the current results. In a few studies, researchers used active control conditions such as pharmacotherapy, progressive relaxation technique, and standard ERP. In addition, those conditions are valuable therapies and may lessen the severity of obsessive–compulsive symptoms. Accordingly, in comparison with these active control groups, ACT may have comparable effects and no apparent superiority. In some studies, ACT has been used alongside other therapies such as narrative therapy and pharmacotherapy. Other researchers used ACT plus ERP as central interventions and ERP as a control condition [39].

To further understand the effects of ACT, subgroup analyses were conducted and showed a significantly large effect size in the active control conditions. A moderate effect size was also found in groups with inactive control conditions, and the inactive conditions were found to have a slightly missed statistical significance. In the forest plot (see Figure 2), it can be seen that the studies of [31,36] showed negligible effect size despite using waitlist control which might be due to the small sample size of the inactive control conditions. As for active conditions, the result was significant statistically (*p* < 0.000). Studies having active conditions favored interventions rather than control, except for the study of Twohig in 2018. They had a statistically insignificant result because ERP, the gold standard, was used as the control condition.

The comprehensive analysis of the studies led to various conclusions. In their meta-analysis and systematic review, [44] found that ACT is “possibly efficacious” in treating OCD. In our current study, we can safely say that ACT is probably effective in treating OCD. There is a higher plausibility because more RCTs and other study designs have been performed to acknowledge the growing presence of ACT in the mental health domain. In addition, our current meta-analysis, even with a small number of studies, provides evidence for the effectiveness of ACT. Precisely, it includes studies with high methodological rigors. Moreover, in line with our systematic review, evidence for using ACT in OCD was drawn not only from RCTs but also from case series and case studies. These studies had evident drawbacks, such as a small sample size or subjective characteristics of the narratives. However, the affirmative results of this current meta-analysis were essential in positing ACT as a conceivable intervention for OCD.

### 4.2. ACT and Waitlist

A study assessed and compared the results of time perspective therapy, ACT, and narrative therapy, controlled with a waitlist group [36]. It headed to a significant diminution of the mean point in obsessive–compulsive symptoms compared to the control group. Consequently, ACT and narrative therapy effectively reduced OCD symptomatology, particularly for patients who forbore exposure treatment. Similarly, Izadi et al., in 2014 [31], compared ACT with a waitlist group and found that there was significant diversity between ACT and waitlist posttreatment. This difference was maintained at follow-up (*p* = 0.01). ACT was also more effective for psychological flexibility, and changes in this particular cognitive process encourage better clinical prognosis in OCD patients. Another RCT comparing ACT with a waitlist group found that ACT improved clinical perfectionism by targeting the dysfunctional core processes rather than being symptom-focused [40]. However, our meta-analysis did not include this study due to different outcome measures.

### 4.3. ACT and SSRIs

A meta-analysis comparing SSRIs with placebo, including 17 studies with 3097 participants, clearly confirmed the efficacy of SSRIs for OCD [35]. Nonetheless, 40 to 60% of patients maintain residual or damaging symptoms, with unfavorable side effects of SSRIs [45]. Additionally, present-day antidepressant treatments require a prolonged waiting phase, ranging from a few weeks to months, until the initiation of symptom recovery. Our meta-analysis results portray the efficacy of ACT + SSRIs or ACT alone, and the effect size for ACT reflects the effectiveness of this intervention over SSRIs. ACT alleviates symptoms of obsessions when compared to using SSRIs alone, even in ACT group therapy. Other studies deliver cross-cultural support for the group-based ACT as a therapy for OCD and as an add-on to SSRIs. It is advantageous for mental health practitioners to treat more patients in one session, thus being able to implement ACT lucratively without compromising its effectiveness [37,38]. Additionally, ACT and conjoined treatment are more helpful for obsessive–compulsive symptoms and experiential avoidance (EA) than SSRIs alone. Similarly, ACT alone reduced EA through acceptance and cognitive defusion. SSRIs were not effective in the short term, which may be due to the nature of the drugs being effective mainly in the long run [46]. This alludes to a study where the combined treatment of Unified Protocol for Transdiagnostic Treatment of Emotional Disorders (UP) and SSRIs showed effectiveness on EA after a one-month follow-up [43].

Furthermore, in one study, 90 OCD patients were treated with ACT, clomipramine (tricyclic antidepressant (TCA)), and combination therapy of ACT and clomipramine [34]. In this study, the attenuation of OCD symptoms in the ACT group and combination group was significantly superior to that in the clomipramine group. Adding clomipramine to ACT to improve the patients’ symptoms does not yield a positive result. Another study by [47], observed a significant decline in OCD symptom severity in ERP + SSRI and ACT + SSRI conditions posttreatment when matched with SSRI-only during follow-up. In addition, psychological inflexibility and thought-restraint maneuvers were significantly reduced in the ACT + SSRI condition posttreatment as well as at follow-up compared to ERP + SSRI and SSRI conditions. Their results brought universal confirmation for treating OCD by using ACT and ERP as an add-on to SSRIs and showcased little differences of change within ACT and ERP. Therefore, it can safely be put forward that adding ACT to SSRIs brings about more symptom reduction than SSRIs alone. For that matter, ACT + SSRI has also been more effective in targeting psychological inflexibility than ERP + SSRI or SSRIs alone.

### 4.4. ACT, ERP, and PRT

Numerous studies support the effectiveness of ERP, which implicates repeated exposure and resistance to internal and external obsessional cues (e.g., dirty objects) without the use of compulsive rituals (e.g., excessive hand washing). A recent review supported that half of the patients following intense ERP treatment, either as monotherapy or in combination with other forms of pharmacotherapy, have milder symptoms [48]. However, most patients who undertake ERP continue to be symptomatic, and some even do not benefit from it. It also has some limitations, such as high dropout rates, the requirement of highly trained therapists, and the reluctance of some patients to adopt ERP [49]. It should be noted that around 25–30% of patients show poor compliance to ERP follow-ups [50]. Different studies conducted by Twohig et al. [39] compared ACT with other active treatments and shed light on whether ACT can be a self-sufficient therapy for OCD. In 2018, Twohig et al [39] stated that ACT did not significantly enhance initial outcomes when attached to an active intervention such as ERP, and change scores were similar between the two groups due to the floor effect. Despite adding ACT to the treatment group, the YBOCS score was low because ERP is relatively effective. Another case study found that four participants improved their psychological flexibility with both ERP and ACT [41]. Overall, this present study could not confirm the non-inferiority of ACT over ERP as there is a scarcity of RCTs comparing the efficacy of ACT against ERP in OCD.

Likewise, an additional study that is not included in our meta-analysis and systematic review due to inadequate data and invariance of sample size assessed changes regarding psychological flexibility during ACT compared to PRT [51]. Differences in treatment were not immediately seen, but steady enhancement was seen for ACT. Across all the three studies of Twohig et al. [30,39,51], it was shown that ACT affected aimed outcomes and change processes. Such patterns designate overall support of the ACT model for improving OCD symptoms, and there is no possible refutation of the underlying psychological flexibility theory in ACT. Another study of good methodological accuracy compared ACT to PRT [30]. In this study, the ACT group showed superior diminutions in OCD severity posttreatment with an ES of 0.77 as well as at a follow-up of 3 months with an ES of 1.10. In addition, psychological flexibility improved considerably from pretreatment to posttreatment in the ACT condition with an ES of 0.59, but not in the PRT group. However, both groups were equivalent in flexibility at follow-up with an ES of 0.22.

### 4.5. ACT in Case Series and Case Studies

In one case study, the successful treatment of a 39-year-old male with OCD using ACT was reported [33]. The patient’s symptoms had not receded despite undergoing several trials of various SSRIs. After eight ACT sessions, a consequent diminution in the OCD symptoms, depression, and anxiety was reported. OCD symptoms diminished by more than 50% over the treatment duration, and these improvements were maintained after three months of follow-up. Another study among five patients having obsessive thoughts assessed the effectiveness of ACT and reckoned that it is a promising tool in the psychological armamentarium [31]. After 10 weekly sessions of one hour, all the five participants showed clinically significant diminutions in OCD symptom severity, which were maintained after a one-month follow-up. Another case series wherein five scrupulosity-based OCD patients underwent eight ACT sessions, carried out in weekly sessions of 1 to 1.5 h duration, found a 74% reduction in compulsions and a 79% reduction in avoided valued behaviors. These improvements were maintained at a three-month follow-up [32]. Similarly, the effectiveness of ACT was investigated during eight weekly sessions of 45 min among eight women diagnosed with OCD [42]. Using the method of visual analysis and improvement percentage, a 60–80% decrease in death anxiety and a 51–60% diminution in obsessive–compulsive symptoms were observed.

### 4.6. Clinical Implications

These findings have several clinical implications. Primarily, the given data favor the practice of ACT in the treatment of OCD, thus putting ACT forward as a valuable therapy for clinicians to remedy an impactful disorder affecting millions of people globally [52]. Given the theoretical evidence, the utmost issue remains in recognizing the ideal situation in which ACT can be applied in a beneficial and ethical approach [53]. Moreover, it was noticed that ACT might influence the quality of life of patients in comparison to conventional CBT techniques. Substantially, CBT comprising exposure maneuvers is yet the first-line psychotherapy for OCD [54]. ACT could be deemed a viable secondary option in cases where CBT with ERP is ineffectual or rejected. With regard to the evidence-based behavioral practice (EBPP), it is recommended to formulate treatment choices based on the available evidence, the therapist’s expertise, and the characteristics of the clients [55]. It alludes to the “dodo bird” hypothesis, concluding that all therapies are of equal prominence, and therapists need to find befitting situations according to the clients’ presentation to administer any therapy. Through its subgroup analysis and review, this present study showcases evidence that may help clinicians find the best available treatment options for OCD. For instance, if the client is resistant or relatively non-compliant with challenging maneuvers, such as exposure practices, the combination of ACT modalities may accentuate participation in treatment [20]. Additionally, this meta-analysis and systematic review also bring forward two particular studies showcasing the use of exposure therapy being compatible with ACT. Intrinsically, it has been stated that ACT might be more similar than different to ERP [49]. ACT has even been referred to as an exposure-based treatment [56]. This meta-analytic review acts as a point of reference for the use of ACT when ERP and traditional CBT are deemed ineffective.

Finally, in this systematic review, several included studies have assessed changes in psychological flexibilities. Numerous studies have concluded that cognitive inflexibility is related to various neurological and behavioral abnormalities in OCD patients [57,58,59]. Moreover, it has been found that changes in cognitive flexibility could justify repetitive thoughts and actions typical of OCD [60]. It has also been observed that OCD patients show more cognitive inflexibility than healthy controls [61,62]. Therefore, having established the occurrence of psychological inflexibility in OCD patients, it can be safely stated that ACT provides a new target for treating OCD which is often neglected in other therapies. Although ACT cannot be a viable full-fledged therapy for OCD yet, it can be an adjunct treatment, especially for SSRIs and ERP. If the results of this study are confirmed in broader samples, the use of ACT for OCD should be encouraged. In a nutshell, ACT remains a “more than probable” treatment for OCD.

## 5. Limitations and Further Directions

Despite shedding light upon the applicability of ACT for OCD, there were also some limitations. Firstly, we included a small number of studies in our meta-analysis, which may have affected our results in some measure and reduced the generalizability of our results. As such, caution should be taken when stating firm findings from these meta-analytic results. We also did not conduct a power analysis to support the statistical power of our meta-analysis. Secondly, our subgroup analysis was insufficient to attain the definite effect due to the small number of studies and its significant small sample size. Thirdly, the studies involved in this meta-analysis had considerable heterogeneity. The studies’ quality, numerous types of population, intervention, duration, and frequency and the OCD severity might influence heterogeneity. Likewise, we did not consider the influence of different frequencies and duration. Finally, only the instant posttreatment outcomes were used to assess the effects of ACT in treating OCD, but a few studies revealed ameliorations in OCD during follow-up sessions.

This meta-analysis and review raise the need for future research. Primarily, more extensive randomized clinical trials should be done on ACT for OCD, focusing on psychological mechanisms such as psychological flexibility. Moreover, more research on ACT from basic sciences and laboratory settings is needed to understand the process of change within the model of ACT. Additionally, studies on the integration of ACT with other behavioral therapies can reinforce the knowledge of the field and capacity to treat OCD. Lastly, for ACT to be a sustainable treatment for OCD, studies with control comparison groups, blind assessment, and bigger sample sizes are needed.

## Figures and Tables

**Table 1:** Characteristics of included studies.

Study and Year	Design	Intervention and Control (N)	Duration	Outcome Measures	Outcome	Dropout
Twohig 2010 [30]	RCT	ACT—36 OCD patients PRT—33	8 sessions weekly ACT or PRT—1 h	YBOCS	ACT posttreatment = 46–56%, PRT posttreatment = 13–18%	10%
Izadi 2012 [31]	Case Series	ACT—5 OCD patients	10 weekly ACT sessions of 1 h	YBOCS	Scores of all subjects dropped below the previously established cutoff score of 18 on the YBOCS scale	NR
Dehlin et al., 2013 [32]	Case Series	ACT—5 Scrupulosity-based OCD patients	8 sessions	YBOCS	Average daily compulsions reduced pretreatment = 25.0, posttreatment = 5.6,	NR
Vakili and Gharraee 2014 [33]	Case Study	ACT—1 OCD patient	8 sessions of ACT with 1, 3, 6 months follow-up	YBOCS	Scores on YBOCS and BAI reduced by 15 points, i.e., 50% from the baseline	NR
Baghooli 2014 [34]	RCT	ACT—25 OCD patients SSRI—25 OCD patients	NR	YBOCS	ACT and combined treatment experienced a greater improvement in obsessive–compulsive symptoms at posttreatment compared to those treated with medication alone, and statistically significant	5.9%
Izadi 2014 [35]	RCT	ACT—25 OCD patients Waitlist—12 patients	10 sessions weekly for 2 h	YBOCS	ACT made significant changes in OCD symptoms	NR
Esfahani 2015 [36]	RCT	ACT—15 OCD patients Waitlist—12 patients	10 sessions weekly, 1 h	YBOCS	ACT is more effective than TPT, NT	NR
Vakili 2015 [37]	RCT	ACT—9 OCD patients SSRI—9 patients	10 weekly sessions	YBOCS	Unlike SSRI alone, ACT and combined treatment led to greater improvement in obsessive–compulsive symptoms and experiential avoidance	1 patient
Rohani 2017 [38]	RCT	ACT + SSRI—23 OCD patients SSRI—23 patients	8 weekly sessions	YBOCS	ACT as a successful adjunct to SSRI	
Twohig 2018 [39]	RCT	ACT + ERP—30 OCD patients ERP—28 patients	16 sessions twice weekly ERP or ACT + ERP	YBOCS	Reduction rate in YBOCS: 70% ACT + ERP 68% ERP	6.9%
Ong 2019 [40]	RCT	ACT—28 OCD patients with clinical perfectionism Waitlist—25 patients	10 sessions of 50 min weekly	Frost Multidimensional Perfectionism Scale	ACT is feasible and efficacious, supporting a shift from symptom-focused to process-based care	35.7%
Thompson 2020 [41]	Case Study	ACT—4 OCD patients	Varied number of sessions of ERP and ACT among patients	YBOCS	Both ACT and ERP can increase psychological flexibility	NR
Davazdahemanni 2020 [42]	Case Series	ACT—8 OCD patients with death anxiety	8 weekly sessions of 45 min	YBOCS	60–80% decrease in death anxiety, 51–60% decrease in OCD symptoms	NR
Zemestani 2021 [43]	RCT	ACT + SSRI—13 OCD patients SSRI—15 OCD	12 individual weekly sessions of 90 min	YBOCS	Psychological inflexibility decreases in ACT + SSRI group	NR

Abbreviations: OCD: obsessive–compulsive disorder; YBOCS: Yale–Brown Obsessive–Compulsive Scale; ERP: exposure–response prevention; ACT: acceptance and commitment therapy; PRT: progressive relaxation technique; SSRI: selective serotonin reuptake inhibitor; RCT: randomized controlled trial; NA: not applicable; NR: not reported.

**Table 2:** Comparison of YBOCS scores between ACT and different controls.

Control Condition Type	Studies (N)	NO. Of Patients	SMDS (95% CI)	*p*-Value (Overall Effect)	I2-Value %	*p*-Value (Heterogeneity)
Overall	8	366	−1.19 (−1.87, −0.51)	0.000	87%	0.004
Active	6	279	−1.38 (−2.248, −0.508)	0.000	90%	0.011
Inactive	2	87	−0.702 (−1.735, −0.332)	0.059	72.1%	0.273

## Data Availability

All the data used in this study can be found in the References.
